# Complete Genome Sequence Analysis of *Pandoraea pnomenusa* Type Strain DSM 16536^T^ Isolated from a Cystic Fibrosis Patient

**DOI:** 10.3389/fmicb.2016.00109

**Published:** 2016-02-08

**Authors:** Yan-Lue Lim, Robson Ee, Delicia Yong, Choo-Yee Yu, Geik-Yong Ang, Kok-Keng Tee, Wai-Fong Yin, Kok-Gan Chan

**Affiliations:** ^1^Division of Genetics and Molecular Biology, Faculty of Science, Institute of Biological Sciences, University of MalayaKuala Lumpur, Malaysia; ^2^Department of Medical Microbiology, Faculty of Medicine, University of MalayaKuala Lumpur, Malaysia

**Keywords:** *Pandoraea pnomenusa*, cystic fibrosis, opportunistic pathogen, complete genome, Single Molecule Real-Time (SMRT) sequencing

## Background

The genus of *Pandoraea* was first proposed in 2000 following the isolation from the sputum of cystic fibrosis patients (Coenye et al., 2000). Five species were initially assigned to the novel genus namely *Pandoraea apista, Pandoraea pulmonicola, Pandoraea pnomenusa, Pandoraea sputorum*, and *Pandoraea norimbergensis* but the description of four new species and another four genomospecies in the subsequent years led to a total of nine species and four genomospecies within the genus of *Pandoraea* (Daneshvar et al., 2001; Anandham et al., 2010; Sahin et al., 2011). The isolation of *Pandoraea* spp. from various environmental samples such as water, sludge, and soils have been reported, but to date, only *P. pnomenusa, P. apista, P. pulmonicola*, and *P. sputorum* were isolated from clinical specimens such as blood, sputum and bronchial fluid of patients with cystic fibrosis or chronic lung diseases (Coenye et al., 2000; Daneshvar et al., 2001; Stryjewski et al., 2003; Han-Jen et al., 2013). Members of *Pandoraea* tend to exhibit broad resistance to ampicillin, extended-spectrum cephalosporins, aztreonam, aminoglycosides, and meropenem but they are sensitive to imipenem (Daneshvar et al., 2001; Stryjewski et al., 2003). However, the clinical significance and prevalence of these multi-drug resistant bacteria among patients with cystic fibrosis or respiratory diseases remained unknown since *Pandoraea* spp. are usually misidentified as *Burkholderia cepacia* complex, *Ralstonia pickettii*, or *Ralstonia paucula* (Segonds et al., 2003). Ambiguity in differentiating between *B. cepacia* complex, *Ralstonia* spp. and *Pandoraea* spp. can be resolved by 16S ribosomal DNA-PCR (Coenye et al., 2001) and *gyrB* gene restriction fragment length polymorphism (Coenye and LiPuma, 2002) but the limited use of molecular typing methods in routine clinical microbiological laboratory has resulted in the underreporting of *Pandoraea* spp. in clinical cases.

The first case of mortality due to *P. pnomenusa* was documented in 2003 whereby the patient developed bacteremia and subsequently died from multiple organ failure after undergoing a bilateral lung transplant operation (Stryjewski et al., 2003). Isolation of *P. pnomenusa* from purulent secretions collected by bronchoalveolar lavage on postoperative day 10 indicated that the lungs could be the source of infection and the cause of fatality was subsequently established through the repeated isolation of *P. pnomenusa* from blood cultures on postoperative day 8 and at the time of death (Stryjewski et al., 2003). In a study conducted by Costello et al. (2011), *P. pnomenusa* was found to be invasive in lung cells expressing cystic fibrosis transmembrane regulator and the species exhibited the greatest level of transepithelial translocation when compared to *P. pulmonicola, P. apista*, and *B. cenocepacia*. Reports of *Pandoraea* -associated bacteraemia and the subsequent isolation of *P. pnomenusa* from blood culture suggest this species may be highly pathogenic (Daneshvar et al., 2001; Stryjewski et al., 2003; Costello et al., 2011). Furthermore, *P. pnomenusa* could trigger the accumulation of pro-inflammatory cytokines, particularly interleukins 8 and 6, which could lead to lung tissue damage. Chronic lung inflammation is one of the main causes of fatality in individuals with cystic fibrosis and hence, the ability of *P. pnomenusa* to elicit the secretion of interleukins 8 and 6 as a part of the host response may serve as one of the mechanisms of pathogenesis for this species (Caraher et al., 2008).

In the last decade, only a few studies have been done to investigate the underlying pathogenic mechanisms of *P. pnomenusa*. In order to gain a better understanding of this species, we studied the complete genome sequence of *P. pnomenusa* type strain DSM 16536^T^ which was isolated from the sputum of a cystic fibrosis patient originating from Edinburgh, United Kingdom. Availability of this genome sequence of *P. pnomenusa* DSM 16536^T^ will serve as a basis for further in-depth analysis of the virulence, pathogenesis and genetics of *P. pnomenusa*.

## Materials and methods

### DNA purification

*P. pnomenusa* DSM 16536^T^, a clinical strain isolated from a cystic fibrosis patient in United Kingdom, was acquired from the German Collection of Microorganisms and Cell cultures (DSMZ). This bacterium was grown aerobically in LB broth at 37°C. Isolation and purification of bacterial genomic DNA was performed with MasterPure DNA Purification Kit (EpiCenter, CA, USA) according to the manufacturer's instruction. The purity, quality and quantity of purified genomic DNA were assessed using NanoDrop 2000 UV-Vis spectrophotometer (Thermo Scientific, MA, USA) and Qubit 2.0 fluorometer (Life Technologies, MA, USA), respectively.

### Genome sequencing and assembly

The sheared genomic DNA of *P. pnomenusa* DSM 16536^T^ was prepared following the “Procedure a Checklist-20kb Template Preparation using BluePippin^TM^ Size Selection” protocol, in which size selection of the constructed SMRTbell templates was performed with a cutoff length of 7kb. Purified and size-selected SMRTbell library was sequenced on four SMRT cells using P5C3 chemistry on Pacific Biosciences (PacBio) Single Molecule, Real-Time (SMRT) RS II instrument. Sub-reads which were generated from the raw sequencing reads after adapter removal were further filtered and mapped prior to *de novo* assembly using Hierarchical Genome Assembly Process (HGAP) version 3. The polished assembly generated was examined for circularity based on the presence of overlapping sequences at both ends of the contig. Location of the overlapping sequence were determined using Gepard dotplot program (Krumsiek et al., 2007). Subsequently, circularization of the contig was performed by removing one of the overlapping ends to generate a blunt-ended circular assembly yielding a complete genome map.

### Genome annotation

Gene prediction and annotation were performed using Rapid Annotation Search Tool (RAST) (Aziz et al., 2008), Rapid Prokaryotic Genome Annotation (Prokka) (Seemann, 2014), and NCBI Prokaryotic Genome Annotation Pipeline (PGAP) based on the Best-placed reference protein set and GeneMarkS+. Additional gene identification was done using KEGG database (Kanehisa et al., 2015), Pathosystem Resource Integration Center (PATRIC) (Wattam et al., 2014), and IMG ER (Markowitz et al., 2009). Both IMG ER and NCBI PGAP were used for the identification of tRNA and rRNA genes. Supplementary annotation of antimicrobial resistance genes was analyzed using ResFinder (Carattoli et al., 2014).

## Results

### Genome characteristics

The genome of *P. pnomenusa* DSM 16536^T^ consists of a single circular chromosome of 5,389,285bp with a mean genome coverage of 244.62-fold and an average GC content of 64.87% (Table 1). No plasmid was found in the genome sequence of this bacterium. A total of 4811 genes was predicted of which 4586 genes were identified as protein coding genes. There are 88 RNA genes consisting of 12 rRNA (4 5S rRNA, 4 16S rRNA, and 4 23S rRNA) and 65 tRNA genes. Besides GenBank (accession number: CP009553.2; http://www.ncbi.nlm.nih.gov/nuccore/CP009553.2), in which the genome sequences data are available in FASTA, annotated GenBank flat file, graphical and ASN.1 formats, functional annotation results of this genome are also accessible through KEGG resources.

**Table 1:** **Genome statistics of *Pandoraea pnomenusa* DSM 16536^*T*^**.

**Attribute**	**Value**	**% of Total**
Genome size (bp)	5,395,224	100.00
DNA coding (bp)	4,748,947	88.02
DNA G+C (bp)	3,499,739	64.87
DNA scaffolds	1	100.00
Total genes	4862	100.00
Protein coding genes	4774	98.19
RNA genes	88	1.81
Pseudo genes	508	10.45
Genes in internal clusters	4042	83.13
Genes with function prediction	4092	84.16
Genes assigned to COGs	3656	75.20
Genes with Pfam domains	4181	85.99
Genes with signal peptides	380	7.82
Genes with transmembrane helices	1195	24.58
CRISPR repeats	1	

The overview of this genome record can be attained from the complete genome directory of KEGG ORGANISMS database (http://www.genome.jp/kegg/catalog/org_list.html) with the organism prefix of ppnm. Further, through the ppnm hyperlink, cross-reference information in the form of protein, and small-molecules interaction network maps (http://www.genome.jp/kegg-bin/show_organism?menu_type=pathway_maps&org=ppnm), BRITE biological systems hierarchical classifications (http://www.genome.jp/kegg-bin/show_organism?menu_type=gene_catalogs&org=ppnm), KEGG modules (http://www.genome.jp/kegg-bin/show_organism?menu_type=pathway_modules&org=ppnm), and whole genome map which can be visualized using genome map browser (http://www.genome.jp/kegg-bin/show_genomemap_top?org_id=ppnm) can be accessed through the subdirectory panel. Further insights to the potential pathogenicity of this clinical strain can also be gained from its genomic information through KEGG Pathogen resource (http://www.genome.jp/kegg/disease/pathogen.html).

### Virulence genes

From the complete genome sequences, various genes which could potentially contributing to pathogenicity were identified using specialty gene annotations in PATRIC server via a combination of few curated virulence factors databases including Virulence Factors Database (VFDB), Victors and PATRIC curated virulence database (PATRIC_VF). A total of 16 virulence factors were identified, namely: Phosphoribosylformylglycinamidine cyclo-ligase (LV28_09200), RNA polymerase sigma factor RpoE (LV28_09850), phosphoenolpyruvate synthase (LV28_01060), RecA protein (LV28_10630), aminase component of anthranilate synthase (LV28_11620), imidazole glycerol phosphate synthase cyclase subunit (LV28_12510), translation elongation factor Tu (LV28_12945; LV28_13105), argininosuccinate synthase (LV28_16050), endonuclease III (LV28_23310), 3-isopropylmalate dehydrogenase (LV28_23930), 3-isopropylmalate dehydrogenase (LV28_23930), RNA-binding protein Hfq (LV28_24345), chorismate synthase (LV28_04475), acetolactate synthase large and small subunit (LV28_04625 and LV28_04620), and chemotaxis protein CheY (LV28_14025). These identified virulence factors are well-characterized virulence determinants in the *Burkholderia pseudomallei* (close phylogenetic neighbor of *P. pnomenusa*) and other pathogens such as *Listeria monocytogens, Actinobacillus pleuropneumoniae, Neisseria meningitis*, and *Shigella flexneri* (Sun et al., 2000; Sheehan et al., 2003; Dons et al., 2004; Pilatz et al., 2006; Sharma and Payne, 2006; Thongboonkerd et al., 2007). Hence, these virulence factors identified could serve as promising gene candidates in studying the underlying pathogenic mechanism of persistent *P. pnomenusa* infection. Furthermore, previous studies also demonstrated targeting against these genes resulted in attenuation suggesting that these predicted virulence factors are potential targets for new therapeutic strategies and vaccines development (Cuccui et al., 2007; Breitbach et al., 2008; Srilunchang et al., 2009).

### Antimicrobial resistance genes

From RAST analysis, a total of 44 genes responsible for antibiotic resistance were identified in the genome. Majority of these genes were previously characterized in various pathogens, including *Pseudomonas aeruginosa* and *Escherichia coli* (Morita et al., 2012; Weatherspoon-Griffin et al., 2014). Full details of these antibiotic resistance genes, including efflux pumps-encoding genes and beta-lactamases-encoding genes (Okusu et al., 1996; Piddock, 2006; Poole, 2011; Poirel et al., 2012; Morita et al., 2014, 2015; Weatherspoon-Griffin et al., 2014; Podnecky et al., 2015) are available in Supplementary Table 1. Furthermore, analysis of antimicrobial resistance gene with ResFinder also revealed the presence of OXA-62, a carbapenem-hydrolyzing oxacillinase, further affirming the finding of Schneider et al. (2006) which demonstrated that OXA-62 is distributed specifically within the *P. pnomenusa* species and producing the imipenem-resistant phenotype.

## Data access

The assembled and annotated genome of *P. pnomenusa* DSM 16536^T^ described in this paper has been deposited in GenBank (accession number of CP009553.2); KEGG database (entry number of T03411) and JGI portal with GOLD ID of Gp0107448 and IMG taxon ID of 2606217238.

## Author contributions

YL, RE, DY, CY, GA, WY perform the experiments and collected the data. KC conceived the idea, obtained the funding and WY managed the finance of the project. YL, RE, DY, CY, GA, WY, and KT prepared the draft, and KC proofread the final draft. All authors approved the final manuscript.

### Conflict of interest statement

The authors declare that the research was conducted in the absence of any commercial or financial relationships that could be construed as a potential conflict of interest.
