# Supplementary material for: Complete Genome Sequence Analysis of Pandoraea pnomenusa Type Strain DSM 16536T Isolated from a Cystic Fibrosis Patient
Source: Front Microbiol. 2016 Feb 8;7:109. doi: 10.3389/fmicb.2016.00109 (PMC4744841; doi:10.3389/fmicb.2016.00109)
Supplement: Supplementary file 1 [file Table1.DOCX]

**Supplementary Table 1.** Antimicrobial resistance genes identified from RAST analysis

| **Feature ID** | **Start** | **Stop** | **Length (bp)** | **Function** | **Subsystem** |
| --- | --- | --- | --- | --- | --- |
| **13 multidrug resistance and tripartite exportation system genes** | | | | | |
| fig\|93220.17.peg.1163 | 1293264 | 1294499 | 1236 | Membrane fusion component of tripartite multidrug resistance system | Multidrug Resistance, Tripartite Systems Found in Gram Negative Bacteria |
| fig\|93220.17.peg.4560 | 4962685 | 4961729 | 957 | Membrane fusion component of tripartite multidrug resistance system | Multidrug Resistance, Tripartite Systems Found in Gram Negative Bacteria |
| fig\|93220.17.peg.4731 | 5152903 | 5151575 | 1329 | Membrane fusion component of tripartite multidrug resistance system | Multidrug Resistance, Tripartite Systems Found in Gram Negative Bacteria |
| fig\|93220.17.peg.949 | 1059666 | 1058599 | 1068 | Membrane fusion component of tripartite multidrug resistance system | Multidrug Resistance, Tripartite Systems Found in Gram Negative Bacteria |
| fig\|93220.17.peg.1164 | 1294537 | 1296087 | 1551 | Inner membrane component of tripartite multidrug resistance system | Multidrug Resistance, Tripartite Systems Found in Gram Negative Bacteria |
| fig\|93220.17.peg.3317 | 3606610 | 3608181 | 1572 | Inner membrane component of tripartite multidrug resistance system | Multidrug Resistance, Tripartite Systems Found in Gram Negative Bacteria |
| fig\|93220.17.peg.4562 | 4964906 | 4962888 | 2019 | Inner membrane component of tripartite multidrug resistance system | Multidrug Resistance, Tripartite Systems Found in Gram Negative Bacteria |
| fig\|93220.17.peg.4730 | 5151582 | 5149996 | 1587 | Inner membrane component of tripartite multidrug resistance system | Multidrug Resistance, Tripartite Systems Found in Gram Negative Bacteria |
| fig\|93220.17.peg.948 | 1058602 | 1056911 | 1692 | Inner membrane component of tripartite multidrug resistance system | Multidrug Resistance, Tripartite Systems Found in Gram Negative Bacteria |
| fig\|93220.17.peg.1162 | 1291649 | 1293202 | 1554 | Outer membrane component of tripartite multidrug resistance system | Multidrug Resistance, Tripartite Systems Found in Gram Negative Bacteria |
| fig\|93220.17.peg.4559 | 4961717 | 4960269 | 1449 | Outer membrane component of tripartite multidrug resistance system | Multidrug Resistance, Tripartite Systems Found in Gram Negative Bacteria |
| fig\|93220.17.peg.4729 | 5149990 | 5148434 | 1557 | Outer membrane component of tripartite multidrug resistance system | Multidrug Resistance, Tripartite Systems Found in Gram Negative Bacteria |
| fig\|93220.17.peg.950 | 1059879 | 1061474 | 1596 | Outer membrane component of tripartite multidrug resistance system | Multidrug Resistance, Tripartite Systems Found in Gram Negative Bacteria |
| **4 beta-lactamases** | | | | | |
| fig\|93220.17.peg.3257 | 3546712 | 3547920 | 1209 | Beta-lactamase (EC 3.5.2.6) | Beta-lactamase |
| fig\|93220.17.peg.4407 | 4795457 | 4796314 | 858 | Beta-lactamase (EC 3.5.2.6) | Beta-lactamase |
| fig\|93220.17.peg.3546 | 3865644 | 3864370 | 1275 | Beta-lactamase class C and other penicillin binding proteins | Beta-lactamase |
| fig\|93220.17.peg.1433 | 1599118 | 1598375 | 744 | Metal-dependent hydrolases of the beta-lactamase superfamily I | Beta-lactamase |
| **23 genes involved in multidrug resistance efflux pumps** | | | | | |
| fig\|93220.17.peg.4552 | 4950168 | 4951481 | 1314 | RND efflux system, membrane fusion protein CmeA | Multidrug Resistance Efflux Pumps |
| fig\|93220.17.peg.538 | 599940 | 598666 | 1275 | RND efflux system, membrane fusion protein CmeA | Multidrug Resistance Efflux Pumps |
| fig\|93220.17.peg.4553 | 4951484 | 4954657 | 3174 | RND efflux system, inner membrane transporter CmeB | Multidrug Resistance Efflux Pumps |
| fig\|93220.17.peg.4689 | 5107133 | 5110312 | 3180 | RND efflux system, inner membrane transporter CmeB | Multidrug Resistance Efflux Pumps |
| fig\|93220.17.peg.537 | 598627 | 595442 | 3186 | RND efflux system, inner membrane transporter CmeB | Multidrug Resistance Efflux Pumps |
| fig\|93220.17.peg.1866 | 2065141 | 2063612 | 1530 | RND efflux system, outer membrane lipoprotein CmeC | Multidrug Resistance Efflux Pumps |
| fig\|93220.17.peg.3807 | 4158850 | 4157279 | 1572 | RND efflux system, outer membrane lipoprotein CmeC | Multidrug Resistance Efflux Pumps |
| fig\|93220.17.peg.4554 | 4954654 | 4956117 | 1464 | RND efflux system, outer membrane lipoprotein CmeC | Multidrug Resistance Efflux Pumps |
| fig\|93220.17.peg.4690 | 5110316 | 5111812 | 1497 | RND efflux system, outer membrane lipoprotein CmeC | Multidrug Resistance Efflux Pumps |
| fig\|93220.17.peg.536 | 595429 | 593873 | 1557 | RND efflux system, outer membrane lipoprotein CmeC | Multidrug Resistance Efflux Pumps |
| fig\|93220.17.peg.4687 | 5105502 | 5104738 | 765 | Transcription repressor of multidrug efflux pump acrAB operon, TetR (AcrR) family | Multidrug Resistance Efflux Pumps |
| fig\|93220.17.peg.540 | 601138 | 602163 | 1026 | Probable transcription regulator protein of MDR efflux pump cluster | Multidrug Resistance Efflux Pumps |
| fig\|93220.17.peg.1028 | 1152989 | 1154368 | 1380 | Multi antimicrobial extrusion protein (Na(+)/drug antiporter), MATE family of MDR efflux pumps | Multidrug Resistance Efflux Pumps,  Riboflavin, FMN and FAD metabolism in plants |
| fig\|93220.17.peg.357 | 399887 | 398526 | 1362 | Multidrug and toxin extrusion (MATE) family efflux pump YdhE/NorM, homolog | Multidrug Resistance Efflux Pumps |
| fig\|93220.17.peg.4114 | 4479836 | 4481365 | 1530 | Multidrug and toxin extrusion (MATE) family efflux pump YdhE/NorM, homolog | Multidrug Resistance Efflux Pumps |
| fig\|93220.17.peg.936 | 1042612 | 1043967 | 1356 | Multi antimicrobial extrusion protein (Na(+)/drug antiporter), MATE family of MDR efflux pumps | Multidrug Resistance Efflux Pumps,  Riboflavin, FMN and FAD metabolism in plants |
| fig\|93220.17.peg.1868 | 2068440 | 2067247 | 1194 | Macrolide-specific efflux protein MacA | Multidrug Resistance Efflux Pumps |
| fig\|93220.17.peg.1867 | 2067228 | 2065153 | 2076 | Macrolide export ATP-binding/permease protein MacB (EC 3.6.3.-) | Multidrug Resistance Efflux Pumps |
| fig\|93220.17.peg.223 | 257620 | 256049 | 1572 | RND efflux system, outer membrane lipoprotein, NodT family | Multidrug Resistance Efflux Pumps |
| fig\|93220.17.peg.4395 | 4772873 | 4774363 | 1491 | RND efflux system, outer membrane lipoprotein, NodT family | Multidrug Resistance Efflux Pumps |
| fig\|93220.17.peg.665 | 752602 | 751049 | 1554 | RND efflux system, outer membrane lipoprotein, NodT family | Multidrug Resistance Efflux Pumps |
| fig\|93220.17.peg.4688 | 5105909 | 5107117 | 1209 | Membrane fusion protein of RND family multidrug efflux pump | Multidrug Resistance Efflux Pumps |
| fig\|93220.17.peg.666 | 755727 | 752599 | 3129 | Acriflavin resistance protein | Multidrug Resistance Efflux Pumps |
